# Alkali‐Metal–Assisted Green‐Solvent Synthesis for In Situ Growth of Perovskite Nanocrystals in Porous Materials

**DOI:** 10.1002/advs.202305880

**Published:** 2024-01-18

**Authors:** Peijun Wang, Bolun Wang, Nan Li, Tong He, Hao Zhang, Lu Zhang, Shengzhong (Frank) Liu

**Affiliations:** ^1^ Dalian National Laboratory for Clean Energy Dalian Institute of Chemical Physics Chinese Academy of Sciences Dalian 116023 China; ^2^ Key Laboratory of Applied Surface and Colloid Chemistry Ministry of Education Shaanxi Engineering Lab for Advanced Energy Technology School of Materials Science and Engineering Shaanxi Normal University Xi'an 710119 China; ^3^ State Key Laboratory of Inorganic Synthesis and Preparative Chemistry College of Chemistry Jilin University Changchun 130012 China; ^4^ School of Chemistry and Chemical Engineering Shaanxi Normal University Xi'an 710119 China; ^5^ Center of Materials Science and Optoelectronics Engineering University of Chinese Academy of Sciences Beijing 100049 China

**Keywords:** alkali metal, green solvent, mesoporous silica, perovskite, zeolite

## Abstract

Inorganic metal halide perovskite CsPbX_3_ (X = I, Br, and Cl) nanocrystals (NCs) are rapidly developed due to their excellent photophysical properties and potential applications in lighting, lasers, and scintillators. However, the materials for growing perovskite NCs are insoluble or hydrolyzed in most green solvents, limiting their further development. Based on rational chemical analysis, an alkali‐metal–assisted green‐solvent synthesis method for in situ growth of CsPbBr_3_ NCs within SAPO‐34 zeolite with bright luminescence is developed. Water is the only solvent used in the whole process. Surprisingly, by the synergistic effect of the channel structure of SAPO‐34 and alkali‐metal ions crystallization regulation, the CsPbBr_3_ NCs embedded in SAPO‐34 assisted by Na^+^ emit bright blue light under ultraviolet illumination, with a 30 nm blue shift comparing to the CsPbBr_3_ NCs assisted by K^+^. Moreover, CsPbBr_3_ NCs can also be grown in mesoporous SiO_2_ SBA‐15 and zeolites including ZSM‐5, AlPO‐5, and SOD, indicating that the method is universal for in situ growth of luminescent perovskite NCs in porous materials. This alkali‐metal–assisted green‐solvent synthesis provides a new strategy for developing high‐quantum–yield, tunable‐emission, and stable perovskite luminescent materials.

## Graphical abstract

A universal synthetic strategy is developed for in situ growth of CsPbBr_3_ NCs in porous materials, utilizing water as the only solvent. The CsPbBr_3_@SAPO‐34 composite synthesized with the assistance of different alkali‐metal ion exhibits significant difference of photoluminescence due to the synergistic effect of the channel structure of SAPO‐34 and alkali‐metal ions crystallization regulation.

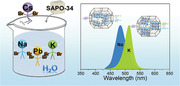

## 1 Introduction

Since Kovalenko et al. first paid attention to the luminescence of inorganic metal halide perovskite CsPbX_3_ (X = I, Br, and Cl) Nanocrystals (NCs) in 2015,^[^ ^1^ ^]^ CsPbX_3_ NCs have been investigated in light‐emitting diodes, lasers, scintillators, liquid‐crystal backlight displays, and so forth in recent years owing to their excellent optical properties.^[^ ^2^, ^3^, ^4^, ^5^, ^6^, ^7^, ^8^, ^9^, ^10^, ^11^, ^12^ ^]^ The emission of CsPbX_3_ NCs can be tuned over the entire visible spectral region (380–780 nm) by adjusting the halides or particle size, with characteristics of high photoluminescence quantum yields and narrow emission line‐widths.^[^ ^1^, ^13^ ^]^ The hot‐injection method, anti‐solvent‐assisted recrystallization method, and microwave‐ or sonication‐assisted crystallization methods have been developed for synthesizing colloidal CsPbX_3_ NCs.^[^ ^14^, ^15^, ^16^, ^17^, ^18^, ^19^, ^20^ ^]^ Because of their small particle size and large specific surface energy, organic ligands are usually introduced to passivate the surface of colloidal CsPbX_3_ NCs. However, problems such as crystal agglomeration and ion migration are inevitable during storage, which would affect their optical properties and even result in quenching.

Additional attempts were made to grow and encapsulate CsPbBr_3_ NCs for promoting their photophysical properties and stability. In general, the encapsulation materials could be classified into several types, including polymers (PMMA, PDMS, polystyrene, etc.),^[^ ^21^, ^22^, ^23^ ^]^ mesoporous silica,^[^ ^24^, ^25^, ^26^, ^27^ ^]^ inorganic salts (Al_2_O_3_, TiO_2_),^[^ ^28^, ^29^ ^]^ metal–organic frameworks,^[^ ^30^, ^31^, ^32^ ^]^ and zeolites.^[^ ^33^, ^34^, ^35^ ^]^ Among them, zeolites are porous aluminosilicates with channels and cavities ranging in size from 0.3 to 1.5 nm connected by [SiO_4_] and [AlO_4_] tetrahedra.^[^ ^36^ ^]^ As composed of pure metal oxides, they are mostly stable against air, oxygen, UV, and thermal stresses. The diverse topological structures, selective pore sizes, and the excellent chemical and thermal stability of porous zeolites provide more possibilities for confining the growth of perovskite NCs and regulating their light emissions. One‐step in situ synthesis is the simplest method and is widely applied for mesoporous SiO_2_ and zeolites.^[^ ^37^, ^38^ ^]^ Perovskite‐DMSO precursor solution and porous materials have been fully mixed and annealed to obtain composites with good luminescence. Zhang et al. proposed an ion‐exchange method to grow CsPbX_3_ and CsPb*_x_* Mn_1−_ *_x_* (Cl, Br)_3_ within zeolite‐Y.^[^ ^11^, ^39^ ^]^ Cs^+^ replaced the metal ions on the framework of zeolite, and then PbX_2_ was introduced to form CsPbX_3_ NCs in the pores of the zeolite. Tong et al. added zeolite into the precursor solution in the hot‐injection process to synthesize CsPbX_3_@zeolite composite and then encapsulated it with PMMA to improve the stability.^[^ ^40^ ^]^ Zhang et al. synthesized CsPbBr_3_ NCs in micro‐mesoporous cross‐linked HSZ ZSM‐5 zeolite by a high‐temperature solid‐phase method, and the optimum calcination temperature was up to 700 °C.^[^ ^33^ ^]^

However, for liquid‐phase methods, limited by the solubility of PbX_2_ and CsX, only a few organic solvents such as DMSO and 1‐octadecence have been used so far to prepare CsPbX_3_ NCs. Their high cost and production of harmful vapors during the annealing process hinder their scale and industrial applications. For solid‐phase methods, high energy consumption and high cost result from the high sublimation and reaction temperature. Rational chemical analysis suggests that alkali‐metal halide would increase the solubility of PbX_2_ in water.^[^ ^41^ ^]^ PbX_2_ could combine with excess halogen ions to form water‐soluble complex ions [PbX_4_]^2−^.

Herein, we developed an alkali‐metal–assisted green‐solvent synthesis method for in situ growth of perovskite NCs in porous materials. This method utilizes only water as a solvent, making it environmentally friendly and energy‐saving. The insoluble PbBr_2_ can be dissolved in water with the assistance of a high concentration of alkali‐metal bromides. We have successfully grown CsPbBr_3_ NCs into SAPO‐34 zeolite with the assistance of KBr or NaBr. By the synergistic effect of the channel structure of SAPO‐34 and alkali‐metal–assisted crystallization regulation, the CsPbBr_3_@SAPO‐34 assisted by K^+^ emits green light under ultraviolet (UV) illumination, while the CsPbBr_3_@SAPO‐34 assisted by Na^+^ shows a blue shift and emits blue light. In contrast, the bright luminescent CsPbBr_3_@SAPO‐34 could not be obtained from perovskite‐DMSO precursor solution. This shows that the superiority of our method, as it allows the substances to diffuse in ionic forms into smaller and intact pore structures of porous materials for in situ growth of perovskite NCs. Moreover, the alkali‐metal–assisted green‐solvent synthesis method is also feasible using mesoporous SiO_2_ SBA‐15 and zeolites including ZSM‐5, AlPO‐5, and SOD, confirming its universality for in situ growth of CsPbBr_3_ NCs into micro‐/meso‐porous materials with bright luminescence.

## 2 Results and Discussion

**Figure 1:**
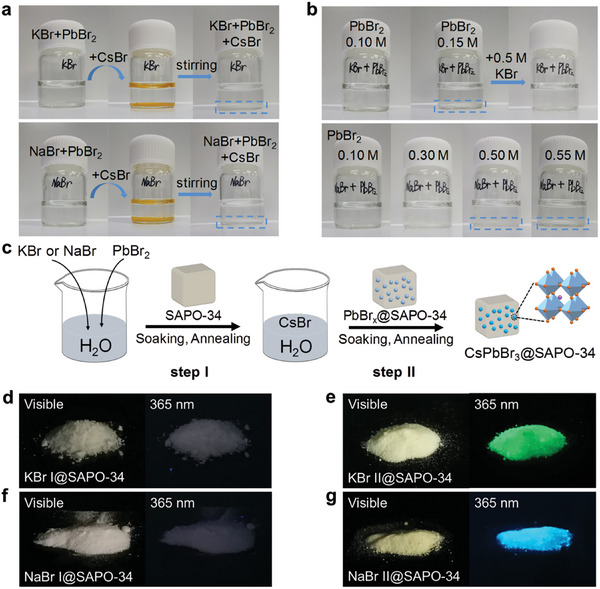
a) Photographs of PbBr_2_ and CsBr dissolved in KBr or NaBr aqueous solution. b) Photographs of different content of PbBr_2_ dissolved in KBr or NaBr aqueous solution. c) Schematic illustration of alkali‐metal–assisted green‐solvent synthesis for CsPbBr_3_@SAPO‐34. d–g) Photographs of the synthesized CsPbBr_3_@SAPO‐34 assisted by KBr or NaBr after step I (b,f) and step II (e,g) under visible illumination and UV illumination at 365 nm.

PbBr_2_ is known to be insoluble in water. However, when combined with excess Br^−^, PbBr_2_ can form a water‐soluble complex ion of [PbBr_4_]^2−^. Therefore a high concentration of alkali‐metal bromide aqueous solution (4.0 m) was prepared to dissolve PbBr_2_ (0.10 m). KBr and NaBr were selected to compare the effects of different alkali‐metal ions on the crystallization of perovskite NCs. Clear PbBr_2_‐KBr and PbBr_2_‐NaBr aqueous solutions were obtained (**Figure** **1**a). However, upon adding CsBr to the PbBr_2_‐KBr/NaBr aqueous solutions, large yellow precipitates immediately formed in both of them, which eventually turned white after stirring. The phenomenon indicates that CsBr could destabilize the PbBr_2_‐KBr/NaBr aqueous solutions, making it impossible to obtain a solution with a specific concentration for one‐step in situ growth of perovskite NCs in porous materials.

To achieve the in situ confined growth of CsPbBr_3_ NCs into zeolite, a two‐step alkali‐metal–assisted green‐solvent synthesis method was developed (Figure 1c). First, a certain concentration of PbBr_2_‐KBr/NaBr aqueous solution was prepared. SAPO‐34 zeolite was immersed into the above solution and sonicated in an ultrasonic washer, so as to exhaust the air and diffuse the solution fully into SAPO‐34 framework. The precipitate was thermal annealed at 100 °C after removing the excess solution. The white intermediate (PbBr*_x_* @SAPO‐34) obtained from the first step was denoted as “KBr I/NaBr I@SAPO‐34.” Second, a CsBr aqueous solution with the same concentration as PbBr_2_ was prepared by dissolving CsBr in H_2_O. The intermediate was soaked in an equal volume of the CsBr aqueous solution and ultrasonically treated. The final composite was obtained after annealing at 100 °C after removing the excess solution. The final pale‐yellow composite was designated “KBr II/NaBr II@SAPO‐34.”

The effect of alkali‐metal ions (K^+^ and Na^+^) on the crystallization of CsPbBr_3_ NCs within SAPO‐34 zeolite is initially demonstrated by examining the solubility of PbBr_2_ in aqueous solutions. Figure 1b shows that a portion of white PbBr_2_ remains undissolved in 4.0 m KBr aqueous solution, when the PbBr_2_ content is increased to 0.15 m. However, the undissolved PbBr_2_ disappeared upon the addition of 0.5 m KBr into the solution. In sharp contrast, the dissolved PbBr_2_ concentration can reach ∽0.50 m in the same concentration (4.0 m) of NaBr aqueous solution. Therefore, the required PbBr_2_ content can be adjusted by modifying the concentration and type of alkali‐metal bromide aqueous solution.

Neither KBr I@SAPO‐34 nor NaBr I@SAPO‐34 glow under UV illumination at 365 nm (Figure 1d,f). We speculate that the estimated tolerance factors (*t*) of less than 0.8 (*t*_K_ ≈ 0.750, *t*_Na_ ≈ 0.669) from the small ionic radii of K^+^ (0.138 nm) and Na^+^ (0.102 nm) make it impossible to form a stable perovskite structure.^[^ ^42^, ^43^ ^]^ However, after introducing CsBr, something shocking and delightful has happened. The CsPbBr_3_@SAPO‐34 composite fabricated with KBr emits green light under 365 nm UV excitation, while the CsPbBr_3_@SAPO‐34 fabricated with NaBr shows blue emission (Figure 1e,g).

**Figure 2:**
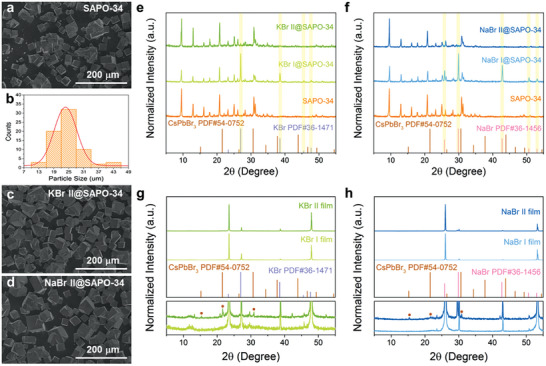
a–d) SEM images of pristine SAPO‐34 zeolite (a), KBr II@SAPO‐34 (c), and NaBr II@SAPO‐34 (d). b) The particle size distribution histograms with Gaussian fitting for pristine SAPO‐34 zeolite. e–h) XRD patterns of composites and films.

SAPO‐34 is a class of zeolite with the CHA‐type octatomic ring pore structure composed of Si, Al, P, and O elements. Scanning electron microscope (SEM) image shows the SAPO‐34 zeolite employed here owns a rectangular morphology with an average particle size of 24.49 µm (**Figure** **2**a,b). With the assistance of KBr or NaBr, the morphologies of the synthesized composites did not change significantly compared with the pristine SAPO‐34 zeolite, as shown in Figure 2c,d. And there are no obvious particles could be observed on the surface of SAPO‐34, which means perovskite NCs grew inside the SAPO‐34 framework. Powder X‐ray diffraction (XRD) patterns of composites are shown in Figure 2e,f. Compared with pristine SAPO‐34 zeolite, KBr I@SAPO‐34 exhibits more characteristic peaks at 26.98°, 38.52°, 45.52°, and 47.68°, which belong to the original KBr (Figure 2e). These peaks weaken sharply and even disappear in the XRD pattern of KBr II@SAPO‐34. This may be due to the high solubility of KBr in water, allowing some KBr crystals in SAPO‐34 pores to dissolve and diffuse out in the CsBr aqueous solution in the step II. No XRD peaks belonging to PbBr_2_ or cubic‐phase CsPbBr_3_ were observed in KBr I@SAPO‐34 or KBr II@SAPO‐34, owing to their low contents within the composites. A similar phenomenon occurred in NaBr I@SAPO‐34 and NaBr II@SAPO‐34 composites (Figure 2f), that is, the additional XRD peaks at 25.86°, 29.94°, 42.84°, 50.72°, and 53.08° in NaBr I@SAPO‐34 consistent with the original NaBr disappear in NaBr II@SAPO‐34. X‐ray photoelectron spectroscopy (XPS) of these composites was performed, as shown in Figure S1 and Table S1, Supporting Information. The atomic ratios of alkali metal K/Na:Br are approximately equal in the four composites. The atomic ratio of K/Na:Pb in step II samples is significantly reduced compared with that in step I. The atomic ratio among Cs:Pb:Br does not match the common compositions of perovskite phases, which is attributed to the different solubility of precursors in water as well as the diffusion and residue of different atoms in the pores of SAPO‐34 during the two steps.

In order to analyze the crystallization from these aqueous solutions, the PbBr_2_‐KBr or PbBr_2_‐NaBr aqueous solutions were dripped directly onto the glass and annealed at 100 °C to form thin film. Then an equal volume of CsBr aqueous solution was dripped onto the film and annealed at 100 °C. The resulting films were measured by XRD (Figure 2g,h). The main components observed in these films were KBr or NaBr. Besides, weak characteristic XRD peaks of CsPbBr_3_ at 15.28°, 21.66°, and 30.88° were observed in KBr II and NaBr II films. It demonstrated that mixing the two‐step solutions and subsequent annealing lead to the formation of CsPbBr_3_ crystals. Additionally, other weak diffraction peaks corresponding to PbBr_2_ and CsBr were also observed. It has been reported that K^+^ and Na^+^ tend to exist at the grain boundaries and surface of perovskite.^[^ ^42^, ^44^, ^45^ ^]^ Thus we denoted the final products as CsPbBr_3_@SAPO‐34 composites according to the original concentration of CsBr:PbBr_2_.

**Figure 3:**
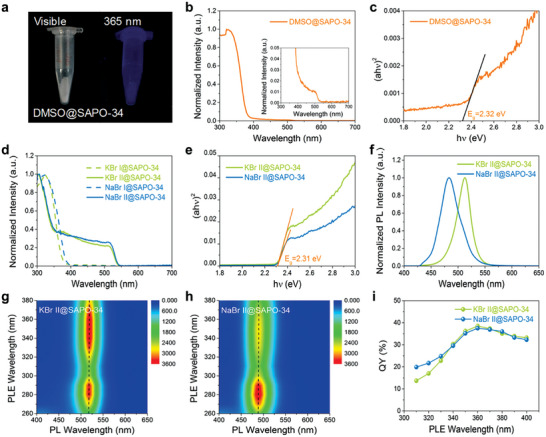
a) Photographs of the one‐step organic‐solvent method synthesized DMSO@SAPO‐34 under visible illumination and UV illumination at 365 nm. b–e) UV–vis absorption spectra and Tauc‐plot curves of DMSO@SAPO‐34, KBr I@SAPO‐34, KBr II@SAPO‐34, NaBr I@SAPO‐34, and NaBr II@SAPO‐34. f) PL spectra of KBr II@SAPO‐34 and NaBr II@SAPO‐34. g,h) Pseudo‐color images of PLE‐PL spectra for KBr II@SAPO‐34 and NaBr II@SAPO‐34, respectively. i) QYs of KBr II@SAPO‐34 and NaBr II@SAPO‐34 under different excitation wavelengths.

To better explain the inspiring luminescence difference of these CsPbBr_3_@SAPO‐34 composites due to the assistance of alkali‐metal ions K^+^ or Na^+^, a series of photophysical characterizations have been performed. The pure CsPbBr_3_@SAPO‐34 composite (denoted as “DMSO@SAPO‐34”) was fabricated as a control for reference from the precursor solution of PbBr_2_ and CsBr in DMSO, the details of which were given in our previously reported work.^[^ ^38^ ^]^ However, DMSO@SAPO‐34 composite with bright luminescence from 365 nm excitation could not be obtained by the one‐step organic‐solvent method, as shown in **Figure** **3**a. There is no obvious absorption band edge of CsPbBr_3_ NCs in the composite (Figure 3b). The locally magnified absorption spectrum shows an extremely weak absorption band edge from ~535 nm attributed to CsPbBr_3_ NCs and corresponding to a bandgap energy of ~2.32 eV (Figure 3c). These results imply that it is difficult to grow perovskite NCs within intact SAPO‐34 zeolite by the one‐step organic‐solvent method. We speculate the reason is that the pore diameter of SAPO‐34 zeolite is only ∽0.68 nm (Figure S2, Supporting Information), which is comparable to the size of a [PbBr_6_] octahedron (~0.6 nm).^[^ ^46^ ^]^ In addition, the connection of several [PbBr_6_] octahedrons and the interaction between PbBr_2_ and DMSO in the perovskite organic precursor solution further hinder the perovskite solution from entering the SAPO‐34 zeolite pores.^[^ ^47^ ^]^ Different from our previous work in which the employed AlPO‐5 zeolite had been ground during the synthesis with damaged pore structure,^[^ ^38^ ^]^ the SAPO‐34 zeolite selected in this work possesses a relatively more‐complete crystal morphology with an intact channel structure, making it difficult for perovskite‐DMSO solution to enter the channels of the SAPO‐34 and diffuse uniformly. This also highlights the advantage of our alkali‐metal–assisted green‐solvent synthesis method to introduce substances in ionic forms and grow perovskites in situ within porous materials with complete crystal structures and smaller pores to form composites for applications in lighting, displays, catalysis fields, and so on.

No perovskite phase was formed in step I, as evidenced by the corresponding UV–vis absorption spectra in Figure 3d for KBr I@SAPO‐34 and NaBr I@SAPO‐34 with no significant absorption band edges in the visible region. A steep absorption band edge appears from ~537 nm in the UV–vis absorption spectra of both KBr II@SAPO‐34 and NaBr II@SAPO‐34 (Figure 3d), which reveals the formation of CsPbBr_3_ NCs within the channels of SAPO‐34 zeolite.^[^ ^48^ ^]^ CsPbBr_3_ is a direct‐bandgap semiconductor.^[^ ^49^ ^]^ Thus the bandgap energy can be derived from a (*αhν*)^2^–*hν* Tauc plot. Here we use absorbance (*a*) instead of the absorption coefficient (*α*). Surprisingly, the two CsPbBr_3_@SAPO‐34 composites, KBr II@SAPO‐34 and NaBr II@SAPO‐34, exhibit the same bandgap energy of 2.31 eV (Figure 3e). This may be caused by the strong light scattering from the big particle size of SAPO‐34 framework. Thus the absorption spectra are more likely to show the absorption features of residual CsPbBr_3_ particles on the surface of zeolite without quantum confinement.

To explain the different photophysical properties of the synthesized CsPbBr_3_@SAPO‐34 composites assisted with K^+^ and Na^+^, photoluminescence (PL) spectra and time‐resolved photoluminescence (TRPL) decays were acquired. As shown in Figure 2f, the PL peak of NaBr II@SAPO‐34 (483 nm) is 30 nm blueshifted in contrast to KBr II@SAPO‐34 (513 nm). The full‐widths at half‐maximum (FWHMs) of NaBr II@SAPO‐34 and KBr II@SAPO‐34 are ∽41 nm and ∽29 nm, respectively. The average exciton lifetime of KBr II@SAPO‐34 fitted with a tri‐exponential function is 31.37 ns, whereas it is only 5.30 ns for NaBr II@SAPO‐34 (Figure S3, Supporting Information). The specific fitting parameters are listed in Table S2, Supporting Information.

The photoluminescence excitation and emission (PLE‐PL) spectra for KBr II@SAPO‐34 and NaBr II@SAPO‐34 excited at different wavelengths from 260 to 380 nm were obtained (Figure 3g,h). The PL peaks of the two composites do not shift obviously with the change of excitation wavelength, which proves that there is only one luminous center in each composite at room temperature. The quantum yields (QYs) of KBr II@SAPO‐34 and NaBr II@SAPO‐34 were measured under different excitation wavelengths from 310 nm to 400 nm with a 10 nm interval (Figure 3i). The QY values excited at 360 nm are the highest, 38.5% for KBr II@SAPO‐34 and 37.5% for NaBr II@SAPO‐34 (Figure S4, Supporting Information).

The variation in emission colors observed in the two composites synthesized by alkali‐metal ions (K^+^ and Na^+^) can potentially be explained by microscopic considerations. The quantum‐confinement–assisted emission occurs when the particle size of the perovskite approaches or is smaller than the exciton Bohr diameter.^[^ ^50^ ^]^ The exciton Bohr diameter of CsPbBr_3_ is ≈7 nm,^[^ ^1^ ^]^ which approximates a connection of 12 layers of [PbBr_6_] octahedrons (denoted as *n* = 12). Limited by the pore size of zeolites, the particle size of CsPbBr_3_ NCs would be smaller. Protesescu et al. synthesized a series of CsPbBr_3_ NCs with different diameters from 11.8 to 3.8 nm that emitted light from green to blue.^[^ ^1^ ^]^ Bohn et al. demonstrated that when the layer number of CsPbBr_3_ NCs was reduced to 6 or less, the NCs showed blue emission, even though the lateral size was 14 ± 4 nm.^[^ ^51^ ^]^ In brief, when the layer number of CsPbBr_3_ NCs in at least one dimension is equal to or less than 6 (*n* ≤ 6), the CsPbBr_3_ NCs are likely to emit blue light. Zhou et al. studied the effect of alkali metals (Li^+^, Na^+^, and K^+^) on quasi‐2D PEA_2_FA_2_Pb_3_Br_10_ perovskite nucleation and growth.^[^ ^52^ ^]^ They found that the incorporation of K^+^ or Na^+^ promoted nucleation of perovskites with smaller layer numbers, resulting in the unannealed perovskite film emitting differently under UV illumination. Cai et al. also demonstrated that NaBr could induce more crystals with small layer numbers in quasi‐2D perovskite for fabricating blue LEDs.^[^ ^53^ ^]^ Zeolite plays a similar role as organic ligands to limit the growth of CsPbBr_3_ NCs along its pore width and depth directions and thus may lead to the formation of perovskite NCs with only a few layers.

**Figure 4:**
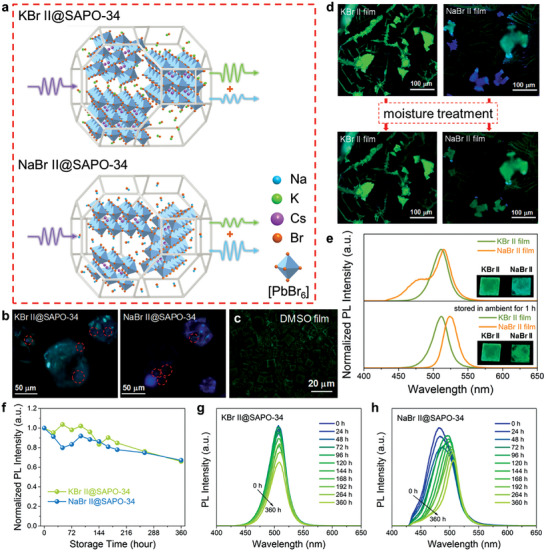
a) Schematic illustration of the influence of K^+^ or Na^+^ on the growth and luminescence of CsPbBr_3_ NCs within SAPO‐34 framework. b) Laser confocal fluorescence micrographs of KBr II@SAPO‐34 and NaBr II@SAPO‐34 composites. c,d) Fluorescence micrographs of DMSO, KBr II, and NaBr II films. e) PL spectra of KBr II and NaBr II films before and after storing in ambient for 1 h. f–h) Time‐dependent PL intensity of KBr II@SAPO‐34 and NaBr II@SAPO‐34 composites stored in ambient.

We speculate that the high concentration of K^+^ or Na^+^ in solution promotes the formation of CsPbBr_3_ NCs in SAPO‐34 with small *n* values (**Figure** **4**a), although the corresponding signals of small *n* values are not observed in the UV–vis absorption spectra because of low content of CsPbBr_3_ NCs loaded into SAPO‐34. In particular, Na^+^ could induce a great increase in proportion of smaller *n* values, leading to blue light emission under UV excitation. The glowing spots in laser confocal fluorescence micrographs at 375 nm excitation show CsPbBr_3_ NCs grew dispersedly within SAPO‐34 (Figure 4b). In addition to cyan light, weak pale blue emission (with dotted circles) could be observed in the same KBr II@SAPO‐34 particle. And weak green light (with dotted circles) could be seen in a NaBr II@SAPO‐34 particle besides blue highlight. These phenomena visually prove that CsPbBr_3_ NCs with various layer numbers could exist in SAPO‐34 framework assisted by K^+^ or Na^+^. The CsPbBr_3_ NCs with different layer numbers in the composites would emit mixed light with wide PL spectra under UV illumination. Thus, the PL curves in Figure 3f are asymmetrical. There are fewer CsPbBr_3_ NCs with small *n* value in KBr II@SAPO‐34, and therefore its luminescence is mainly green with a small amount of blue light, and the corresponding PL spectrum is wider to the left. Similarly, there is some green light in the luminescence of NaBr II@SAPO‐34, thus the right branch of its PL spectrum is wider.

In order to study the effects of K^+^ and Na^+^ on the crystallization and growth of CsPbBr_3_ NCs, pristine DMSO, KBr II, and NaBr II films were characterized. In contrast to the formation of square CsPbBr_3_ crystals synthesized by DMSO precursor solution (Figure 4c), fluorescence micrographs reveal a combination of dendritic and granular growth patterns for CsPbBr_3_ synthesized with KBr or NaBr (Figure 4d). The dendritic crystals in both KBr II and NaBr II films emit green light. However, the granular crystals in NaBr II film show blue and cyan emission, which differs from the green light emitted by granular crystals in KBr II film. Focusing on these granular crystals (Figure S5, Supporting Information), it becomes evident that only scattered points within the particle emit light, rather than the entire particle glowing. The granular crystals mainly consist of KBr or NaBr crystals, and encase small size of CsPbBr_3_ NCs within their matrices.^[^ ^41^ ^]^ A large number of alkali‐metal bromides could interrupt the continuous growth of CsPbBr_3_ crystals. Upon exposure to moisture, the dendritic crystals in NaBr II film decompose, while the granular crystals partially dissolve and then recrystallize, resulting in a shift in luminescence from blue to green, as shown in Figure 4d. In contrast, the crystals in KBr II film exhibit no significant decomposition. It is due to the higher sensitivity of NaBr to moisture and its tendency to deliquesce more easily.

Figure 4e shows the PL spectra of freshly prepared KBr II and NaBr II films, exhibiting peaks at 511 and 515 nm, respectively. And there was a shoulder peak on the left side of the PL curve for NaBr II film. Comparing with KBr II@SAPO‐34 and NaBr II@SAPO‐34 composite, it can be observed that the PL peaks of both films experience a redshift, indicating the quantum‐confined effect of SAPO‐34 on CsPbBr_3_ NCs. Upon exposure to ambient conditions (RH 52%) for ≈1 h, the luminescence of NaBr II film transitions from blue‐green to green, as insets in Figure 4e show. And the corresponding PL peak redshifts to 524 nm, demonstrating the moisture instability of NaBr II film. Conversely, the luminescence and PL peak of KBr II film did not change significantly. These phenomena reflect the effects of different alkali‐metal ions on the crystallization and stability of CsPbBr_3_. The stability in ambient of KBr II@SAPO‐34 and NaBr II@SAPO‐34 composites were recorded (Figure 4f–h). The PL intensity of KBr II@SAPO‐34 decreased to 65.89% of its initial intensity after storing in ambient (RH 40–60%) for 360 h, without obvious peak shift (Figure 4g). The PL intensity of NaBr II@SAPO‐34 decreased to 67.12% of its initial intensity after 360 h storage in ambient. Figure 4h shows the PL curves of NaBr II@SAPO‐34 red shifted from 481 to 506 nm, accompanied by narrowed FWHMs values. These results suggest that SAPO‐34 zeolite quantum confined the growth of CsPbBr_3_ NCs and stabilized them to some extent.

**Figure 5:**
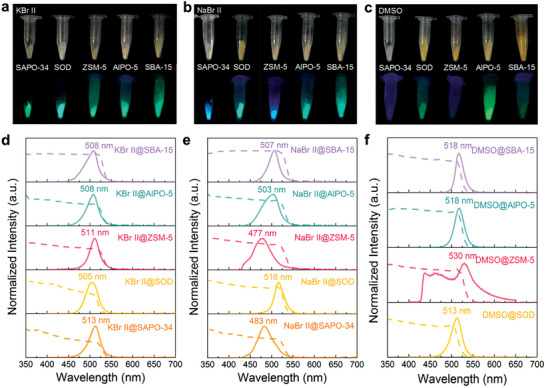
a–c) Photographs of synthesized composites with different micro‐/meso‐porous materials by KBr‐/NaBr‐assisted green‐solvent method and one‐step organic‐solvent method under visible illumination and UV illumination at 365 nm. d–f) UV–vis absorption (dashed lines) and PL (solid lines) spectra of synthesized composites with different micro‐/meso‐porous materials by KBr‐/NaBr‐assisted green‐solvent method and one‐step organic‐solvent method.

In order to demonstrate the universality of alkali‐metal–assisted green‐solvent synthesis method for in situ growth of luminous perovskite@porous material composites, we have also successfully grown CsPbBr_3_ NCs in micro‐/meso‐porous materials including mesoporous silica SBA‐15, zeolites ZSM‐5, AlPO‐5, and SOD. Bright luminescence under UV excitation was observed in these composites (**Figure** **5**a,b). Among them, the CsPbBr_3_ NCs in ZSM‐5 zeolite show the same light emission phenomenon and similar photophysical properties as the CsPbBr_3_ NCs in SAPO‐34 zeolite. Under UV illumination at 365 nm, KBr II@ZSM‐5 emits green light (511 nm), while NaBr II@ZSM‐5 emits blue light (477 nm) (Figure 5d,e). However, the reference DMSO@ZSM‐5 composite synthesized by the one‐step organic‐solvent method does not glow conspicuously under UV illumination, even though the composite changes from the white color of zeolite to yellow (Figure 5c). Also, the absorption spectrum of DMSO@ZSM‐5 shows a clear absorption band edge of CsPbBr_3_ NCs (Figure 5f), while the PL spectrum shows that there are two PL peaks at ∽463 and 530 nm. The PL peak at ∽463 nm derives from the residual template agent inside the ZSM‐5 framework during calcination due to the intact rectangular structure of ZSM‐5 zeolite (Figure S6b, Supporting Information). The residual template agent also hinders the diffusion of perovskite‐organic precursor solution within the ZSM‐5 pores. Thus perovskite NCs tend to grow into larger particles on the surface of ZSM‐5 zeolite with weak PL intensity at ∽530 nm. In contrast, with the assistance of KBr or NaBr, each kind of particle can easily diffuse into the deep pores of ZSM‐5 zeolite in ionic forms in water. Therefore, the PL intensity of CsPbBr_3_ NCs in the KBr II@ZSM‐5 or NaBr II@ZSM‐5 is so intense that the PL peak of ZSM‐5 zeolite itself is negligible.

In contrast to SAPO‐34 and ZSM‐5, the CsPbBr_3_ NCs grown in SBA‐15, AlPO‐5, and SOD with the assistance of either K^+^ or Na^+^ both emit green light at 365 nm excitation. We attribute this to the irregular shapes and incomplete channel structures of these porous materials caused by grinding during synthesis (Figure S6, Supporting Information), which contribute to the larger *n* values of CsPbBr_3_ NCs and the reduced proportion of small‐*n*–value perovskites. This is similar to the (OL)_2_A*_n_*_−1_B*_n_* X_3_ *_n_*_+1_ perovskite fabricated by the precursor solution with *n* > 4, in which large‐*n*–value perovskites dominate.^[^ ^54^, ^55^ ^]^ In addition, composites with bright luminescence fabricated from the perovskite DMSO precursor solution in the three porous materials, namely, AlPO‐5, SBA‐15, and SOD, were successfully prepared (Figure 5c).

## 3 Conclusion

In summary, we have developed an alkali‐metal–assisted green‐solvent method for in situ growth of luminous perovskite NCs in porous materials and successfully prepared CsPbBr_3_@SAPO‐34 with bright luminescence. Throughout the entire synthesis process, water is the only solvent used, which is environmentally friendly and energy‐saving. The highlight of this method is the use of a high concentration of alkali‐metal bromide to both induce the formation of water‐soluble complex ion [PbBr_4_]^2−^ in H_2_O and promote them to easily enter the porous materials with complete crystal structures and smaller pores to form composites. In addition, the different alkali‐metal ions (K^+^ and Na^+^) have different effects on the crystallization and photophysical properties of CsPbBr_3_ NCs within porous materials. We have also synthesized composites with bright emission in mesoporous silica SBA‐15, as well as zeolites such as ZSM‐5, AlPO‐5, and SOD, which demonstrate the universality of this alkali‐metal–assisted green‐solvent synthesis method. Compared to the DMSO@SAPO‐34 and DMSO@ZSM‐5 without bright luminescence obtained by the one‐step organic‐solvent method, our method shows the advantage of allowing substances to enter and uniformly diffuse into smaller and deeper pores of porous materials in ionic forms for growing perovskite NCs with bright luminescence. The novel perovskite@porous material composites have potential applications in lighting, displays, catalysis, energy conversion, and other fields. More types of perovskite@porous materials and more‐effective characterization techniques need to be further explored.

## Conflict of Interest

The authors declare no conflict of interest.

## Supporting information

Supporting Information

## Data Availability

The data that support the findings of this study are available from the corresponding author upon reasonable request.

## References

[1] L. Protesescu , S. Yakunin , M. I. Bodnarchuk , F. Krieg , R. Caputo , C. H. Hendon , R. X. Yang , A. Walsh , M. V. Kovalenko , Nano Lett. 2015, 15, 3692. 25633588 10.1021/nl5048779 PMC4462997

[2] S. Chen , J. Lin , S. Zheng , Y. Zheng , D. Chen , Adv. Funct. Mater. 2023, 33, 2213442.

[3] X. Yang , C. Valenzuela , X. Zhang , Y. H. Chen , Y. Z. Yang , L. Wang , W. Feng , Matter 2023, 6, 1278.

[4] Y. Ru , B. Zhang , X. Yong , L. Sui , J. Yu , H. Song , S. Lu , Adv. Mater. 2023, 35, 2207265. 10.1002/adma.202207265 36408928

[5] Y. Li , Y. Xu , Y. Yang , Z. Jia , H. Tang , J. B. Patel , Q. Lin , Adv. Opt. Mater. 2023, 11, 2300169.

[6] L. Wang , K. Fu , R. Sun , H. Lian , X. Hu , Y. Zhang , Nano‐Micro Lett. 2019, 11, 52. 10.1007/s40820-019-0283-z PMC7770729 34138025

[7] M. Jiang , Z. Hu , Z. Liu , Z. Wu , L. K. Ono , Y. Qi , ACS Energy Lett. 2019, 4, 2731.

[8] C.‐Y. Huang , H. Li , Y. Wu , C.‐H. Lin , X. Guan , L. Hu , J. Kim , X. Zhu , H. Zeng , T. Wu , Nano‐Micro Lett. 2022, 15, 16. 10.1007/s40820-022-00983-6 PMC9800676 36580150

[9] X. Li , Y. Wang , H. Sun , H. Zeng , Adv. Mater. 2017, 29, 1701185. 10.1002/adma.201701185 28758693

[10] W. Ma , T. Jiang , Z. Yang , H. Zhang , Y. Su , Z. Chen , X. Chen , Y. Ma , W. Zhu , X. Yu , H. Zhu , J. Qiu , X. Liu , X. Xu , Y. M. Yang , Adv. Sci. 2021, 8, 2003728. 10.1002/advs.202003728 PMC8336613 34075729

[11] J.‐Y. Sun , F. T. Rabouw , X.‐F. Yang , X.‐Y. Huang , X.‐P. Jing , S. Ye , Q.‐Y. Zhang , Adv. Funct. Mater. 2017, 27, 1704371.

[12] S. Yakunin , L. Protesescu , F. Krieg , M. I. Bodnarchuk , G. Nedelcu , M. Humer , G. De Luca , M. Fiebig , W. Heiss , M. V. Kovalenko , Nat. Commun. 2015, 6, 9056. 10.1038/ncomms9056 PMC4560790 26290056

[13] W.‐G. Lu , C. Chen , D. Han , L. Yao , J. Han , H. Zhong , Y. Wang , Adv. Opt. Mater. 2016, 4, 1732.

[14] J. Pan , Y. Shang , J. Yin , M. De Bastiani , W. Peng , I. Dursun , L. Sinatra , A. M. El‐Zohry , M. N. Hedhili , A.‐H. Emwas , O. F. Mohammed , Z. Ning , O. M. Bakr , J. Am. Chem. Soc. 2018, 140, 562. 29249159 10.1021/jacs.7b10647

[15] Y. Wang , X. Li , S. Sreejith , F. Cao , Z. Wang , M. C. Stuparu , H. Zeng , H. Sun , Adv. Mater. 2016, 28, 10637. 27714913 10.1002/adma.201604110

[16] J. Shamsi , Z. Dang , P. Bianchini , C. Canale , F. Di Stasio , R. Brescia , M. Prato , L. Manna , J. Am. Chem. Soc. 2016, 138, 7240. 27228475 10.1021/jacs.6b03166 PMC4995059

[17] X. Li , Y. Wu , S. Zhang , B. Cai , Y. Gu , J. Song , H. Zeng , Adv. Funct. Mater. 2016, 26, 2435.

[18] F. Zhang , H. Zhong , C. Chen , X.‐G. Wu , X. Hu , H. Huang , J. Han , B. Zou , Y. Dong , ACS Nano 2015, 9, 4533. 25824283 10.1021/acsnano.5b01154

[19] Y. Tong , B. J. Bohn , E. Bladt , K. Wang , P. Müller‐Buschbaum , S. Bals , A. S. Urban , L. Polavarapu , J. Feldmann , Angew. Chem., Int. Ed. 2017, 56, 13887. 10.1002/anie.201707224 28834091

[20] Q. Pan , H. Hu , Y. Zou , M. Chen , L. Wu , D. Yang , X. Yuan , J. Fan , B. Sun , Q. Zhang , J. Mater. Chem. C 2017, 5, 10947.

[21] S. Pathak , N. Sakai , F. W. R. Rivarola , S. D. Stranks , J. Liu , G. E. Eperon , C. Ducati , K. Wojciechowski , J. T. Griffiths , A. A. Haghighirad , A. Pellaroque , R. H. Friend , H. J. Snaith , Chem. Mater. 2015, 27, 8066.

[22] L. Shi , L. Meng , F. Jiang , Y. Ge , F. Li , X.‐G. Wu , H. Zhong , Adv. Funct. Mater. 2019, 29, 1903648.

[23] Z. Gu , Z. Huang , X. Hu , Y. Wang , L. Li , M. Li , Y. Song , ACS Appl. Mater. Interfaces 2020, 12, 22157. 32312039 10.1021/acsami.0c04131

[24] V. Malgras , J. Henzie , T. Takei , Y. Yamauchi , Angew. Chem., Int. Ed. 2018, 57, 8881. 10.1002/anie.201802335 29901830

[25] Q. Zhang , B. Wang , W. Zheng , L. Kong , Q. Wan , C. Zhang , Z. Li , X. Cao , M. Liu , L. Li , Nat. Commun. 2020, 11, 31. 31911597 10.1038/s41467-019-13881-0 PMC6946649

[26] M. He , S. Liu , L. Ding , Z. Zhang , J. Liu , W. Xiang , X. Liang , J. Am. Ceram. Soc. 2019, 102, 930.

[27] Q. Dong , B. Tian , W. Zhang , L. He , Colloids Surf., A 2022, 648, 129258.

[28] A. Kojima , M. Ikegami , K. Teshima , T. Miyasaka , Chem. Lett. 2012, 41, 397.

[29] Z.‐J. Li , E. Hofman , J. Li , A. H. Davis , C.‐H. Tung , L.‐Z. Wu , W. Zheng , Adv. Funct. Mater. 2017, 28, 1704288.

[30] J. Ren , T. Li , X. Zhou , X. Dong , A. V. Shorokhov , M. B. Semenov , V. D. Krevchik , Y. Wang , Chem. Eng. J. 2019, 358, 30.

[31] Z.‐C. Kong , J.‐F. Liao , Y.‐J. Dong , Y.‐F. Xu , H.‐Y. Chen , D.‐B. Kuang , C.‐Y. Su , ACS Energy Lett. 2018, 3, 2656.

[32] C. Zhang , B. Wang , W. Li , S. Huang , L. Kong , Z. Li , L. Li , Nat. Commun. 2017, 8, 1138. 29089491 10.1038/s41467-017-01248-2 PMC5663915

[33] Y. Zhang , L. Han , B. Li , Y. Xu , Chem. Eng. J. 2022, 437, 135290.

[34] L. Han , Y. Han , J. Wu , X. Zhang , Z. Wang , Y. Xu , Mater. Chem. Front. 2021, 5, 7843.

[35] J. Y. Kim , K. I. Shim , J. W. Han , J. Joo , N. H. Heo , K. Seff , Adv. Mater. 2020, 32, 2001868. 10.1002/adma.202001868 32686270

[36] C. Martínez , A. Corma , in Comprehensive Inorganic Chemistry II, 2nd ed. (Eds: J. Reedijk , K. Poeppelmeier ), Elsevier, Amsterdam 2013, pp. 103–131.

[37] D. N. Dirin , L. Protesescu , D. Trummer , I. V. Kochetygov , S. Yakunin , F. Krumeich , N. P. Stadie , M. V. Kovalenko , Nano Lett. 2016, 16, 5866. 27550860 10.1021/acs.nanolett.6b02688 PMC5799875

[38] P. Wang , B. Wang , Y. Liu , L. Li , H. Zhao , Y. Chen , J. Li , S. Liu , K. Zhao , Angew. Chem., Int. Ed. 2020, 59, 23100. 10.1002/anie.202011203 32889779

[39] S. Ye , J.‐Y. Sun , Y.‐H. Han , Y.‐Y. Zhou , Q.‐Y. Zhang , ACS Appl. Mater. Interfaces 2018, 10, 24656. 29979021 10.1021/acsami.8b08342

[40] Y. Tong , M. Jin , Y. Chen , Y. Zhao , H. Yang , Q. Wang , L. Zhai , X. Liang , W. Xiang , J. Mater. Chem. C 2021, 9, 2530.

[41] L. Chen , M. He , L. Li , S. Yuan , A. Chen , M. Chen , Y. Wang , L. Sun , L. Wei , T. Zhang , Q. Li , Q. Zhang , Chem. Eng. J. 2022, 450, 138279.

[42] A. Kausar , A. Sattar , C. Xu , S. Zhang , Z. Kang , Y. Zhang , Chem. Soc. Rev. 2021, 50, 2696. 33409511 10.1039/d0cs01316a

[43] H. Huang , M. I. Bodnarchuk , S. V. Kershaw , M. V. Kovalenko , A. L. Rogach , ACS Energy Lett. 2017, 2, 2071. 28920080 10.1021/acsenergylett.7b00547 PMC5594444

[44] M. Abdi‐Jalebi , Z. Andaji‐Garmaroudi , A. J. Pearson , G. Divitini , S. Cacovich , B. Philippe , H. Rensmo , C. Ducati , R. H. Friend , S. D. Stranks , ACS Energy Lett. 2018, 3, 2671. 30701195 10.1021/acsenergylett.8b01504 PMC6344034

[45] M. Abdi‐Jalebi , M. Pazoki , B. Philippe , M. I. Dar , M. Alsari , A. Sadhanala , G. Divitini , R. Imani , S. Lilliu , J. Kullgren , H. Rensmo , M. Grätzel , R. H. Friend , ACS Nano 2018, 12, 7301. 29953817 10.1021/acsnano.8b03586

[46] E. R. Dohner , E. T. Hoke , H. I. Karunadasa , J. Am. Chem. Soc. 2014, 136, 1718. 24422494 10.1021/ja411045r

[47] M. Jung , S.‐G. Ji , G. Kim , S. I. Seok , Chem. Soc. Rev. 2019, 48, 2011. 30604792 10.1039/c8cs00656c

[48] F. Yan , S. T. Tan , X. Li , H. V. Demir , Small 2019, 15, 1902079. 10.1002/smll.201902079 31650694

[49] J. Di , H. Li , J. Su , H. Yuan , Z. Lin , K. Zhao , J. Chang , Y. Hao , Adv. Sci. 2022, 9, e2103482. 10.1002/advs.202103482 PMC8805584 34761562

[50] J. N. Yang , T. Chen , J. Ge , J. J. Wang , Y. C. Yin , Y. F. Lan , X. C. Ru , Z. Y. Ma , Q. Zhang , H. B. Yao , J. Am. Chem. Soc. 2021, 143, 19928. 34766754 10.1021/jacs.1c09948

[51] B. J. Bohn , Y. Tong , M. Gramlich , M. L. Lai , M. Döblinger , K. Wang , R. L. Z. Hoye , P. Müller‐Buschbaum , S. D. Stranks , A. S. Urban , L. Polavarapu , J. Feldmann , Nano Lett. 2018, 18, 5231. 29990435 10.1021/acs.nanolett.8b02190

[52] Z. Guo , Y. Zhang , B. Wang , L. Wang , N. Zhou , Z. Qiu , N. Li , Y. Chen , C. Zhu , H. Xie , T. Song , L. Song , H. Xue , S. Tao , Q. Chen , G. Xing , L. Xiao , Z. Liu , H. Zhou , Adv. Mater. 2021, 33, 2102246. 10.1002/adma.202102246 34396606

[53] W. Cai , M. U. Ali , P. Liu , M. He , C. Zhao , Z. Chen , Y. Zang , M.‐C. Tang , H. Meng , H. Fu , G. Wei , H.‐L. Yip , Adv. Sci. 2022, 9, e2200393. 10.1002/advs.202200393 PMC9284168 35561063

[54] J. Liu , J. Leng , K. Wu , J. Zhang , S. Jin , J. Am. Chem. Soc. 2017, 139, 1432. 28094931 10.1021/jacs.6b12581

[55] L. N. Quan , M. Yuan , R. Comin , O. Voznyy , E. M. Beauregard , S. Hoogland , A. Buin , A. R. Kirmani , K. Zhao , A. Amassian , D. H. Kim , E. H. Sargent , J. Am. Chem. Soc. 2016, 138, 2649. 26841130 10.1021/jacs.5b11740

