# Supplementary material for: Alkali‐Metal–Assisted Green‐Solvent Synthesis for In Situ Growth of Perovskite Nanocrystals in Porous Materials
Source: Adv Sci (Weinh). 2024 Jan 18;11(12):2305880. doi: 10.1002/advs.202305880 (PMC10966523; doi:10.1002/advs.202305880)
Supplement: Supplementary file 1 — Supporting Information [file ADVS-11-2305880-s001.pdf]

ADVANCED SCIENCE
Open Access

Supporting Information

for *Adv. Sci.*, DOI 10.1002/advs.202305880

Alkali-Metal–Assisted Green-Solvent Synthesis for In Situ Growth of Perovskite Nanocrystals in Porous Materials

Peijun Wang, Bolun Wang, Nan Li, Tong He, Hao Zhang, Lu Zhang and Shengzhong (Frank) Liu**

Alkali-Metal-Assisted Green-Solvent Synthesis for In Situ Growth of Perovskite Nanocrystals in Porous Materials

Peijun Wang[#1, 2, 5], Bolun Wang[#3], Nan Li[2], Tong He[4], Hao Zhang[2], Lu Zhang*[2], Shengzhong (Frank) Liu*[1, 2, 5]

[1]Dalian National Laboratory for Clean Energy, Dalian Institute of Chemical Physics, Chinese Academy of Sciences, Dalian 116023, China

E-mail: szliu@dicp.ac.cn

[2]Key Laboratory of Applied Surface and Colloid Chemistry, Ministry of Education, Shaanxi Engineering Lab for Advanced Energy Technology, School of Materials Science and Engineering, Shaanxi Normal University, Xi'an 710119, China

E-mail: luzhang@snnu.edu.cn

[3]State Key Laboratory of Inorganic Synthesis and Preparative Chemistry, College of Chemistry, Jilin University, Changchun 130012, China

[4]School of Chemistry and Chemical Engineering, Shaanxi Normal University, Xi'an 710119, China

[5]Center of Materials Science and Optoelectronics Engineering, University of Chinese Academy of Sciences, Beijing 100049, China

[#]These authors contributed equally to this work.

Experimental section

Materials:

Cesium bromide (CsBr, ≥ 99.9%) was acquired from Xi'an Polymer Light Technology Corp. Lead bromide ($PbBr_2$, 99.0%) was purchased from Shanghai Aladdin Biochemical Technology Co. Ltd. Potassium bromide (KBr, ≥ 99.0%) was supplied by Guangdong Guanghua Sci-Tech Co. Ltd. And Sodium bromide (NaBr, ≥ 99.0%) was purchased from Sinopharm Chemical Reagent Co. Ltd. DImethyl sulfoxide (DMSO, ≥ 99.8%) was supplied by Alfa Aesar (China). The chemicals were used without further purifications.

Synthesis of $CsPbBr_3$@SAPO-34:

Step I: 0.0367 g $PbBr_2$ was dissolved into 1 mL ultrapure water with 4 M KBr or NaBr. 0.1 g SAPO-34 zeolite was immersed into 400 μL $PbBr_2$-KBr/NaBr aqueous solution and sonicated in an ultrasonic washer for 20 min, so as to exhaust the air in the pores and diffuse the solution fully. The precipitate was thermal annealed at 100ºC for 15 min after removing the excess solution. The white intermediate ($PbBr_x$@SAPO-34) attained from the first step was denoted as “KBr I/NaBr I@SAPO-34”.

Step II: a CsBr aqueous solution with the same concentration as $PbBr_2$ was prepared by dissolved 0.0213 g CsBr into 1 mL ultrapure water. The KBr I/NaBr I@SAPO-34 was soaked into 400 μL CsBr aqueous solution and ultrasonic treated for 20 min. The final composite was obtained after annealing at 100ºC for 20 min after removing the excess solution. And “KBr II/NaBr II@SAPO-34” was assigned to the final composite $CsPbBr_3$@SAPO-34 with pale yellow.

The $CsPbBr_3$@porous materials were synthesized by the same processes of $CsPbBr_3$@SAPO-34.

Film Preparation:

The clear glass was UV-O_3 treated for 15 min. 60 μL $PbBr_2$-KBr/NaBr aqueous solution was dripped onto the glass and annealed for 5 min at 100°C. Then 60 μL CsBr aqueous solution was dripped and annealed for 10 min at 100°C. The KBr II or NaBr II film was prepared.

Measurement and characterization:

XRD measurement was carried out in a θ-2θ configuration with a scanning interval of 2θ between 5° and 60° on DX2700BH (X-ray source: Cu Kα, λ = 1.54 Å). SEM images and EDS were conducted on a field emission scanning electron microscopy (FE-SEM), SU-8020 from Hitachi. XPS were performed by ESCALAB 250Xi X-ray photoelectron spectrometer from Thermo Fisher Scientific. UV-Vis absorption spectra were acquired on a PerkinElmer

UV-Lambda 1050 instrument. Steady-state PL and TRPL spectra (excitation at 375 nm with a 430 nm filter) were measured with PicoQuant FT-300. QYs were obtained by a HAMAMATSU Quantaurus-QY C9920-02G. PLE-PL spectra were measured by a fluorescence spectrophotometer F7100 from Hitachi with a 380 nm filter. The pore width distribution of zeolite was demonstrated by micromeritics ASAP 2020 HD88. Laser confocal fluorescence micrographs were obtained by a OLYMPUS IX73 at 375 nm excitation. Fluorescence micrographs were acquired by a OLYMPUS BX53M with a mercury lamp (excitation wavelength 330-385 nm).

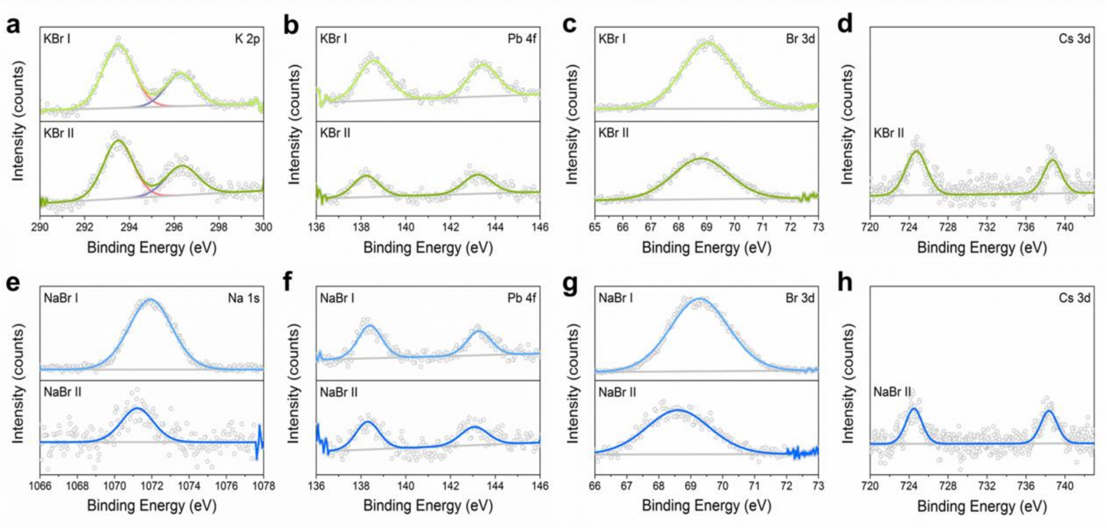

Figure S1. High-resolution X-ray photoelectron spectra of K 2p, Na 1s, Pb 4f, Br 3d, Cs 3d in KBr I@SAPO-34, KBr II@SAPO-34, NaBr I@SAPO-34 and NaBr II@SAPO-34.

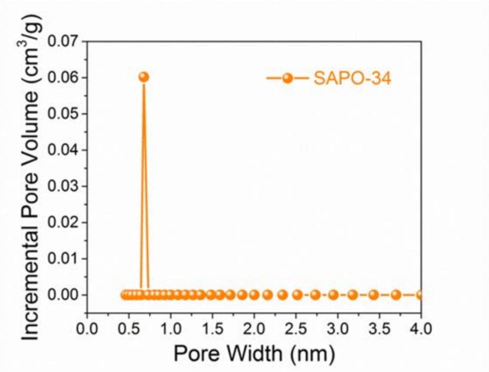

Figure S2. Pore width distribution of SAPO-34 zeolite.

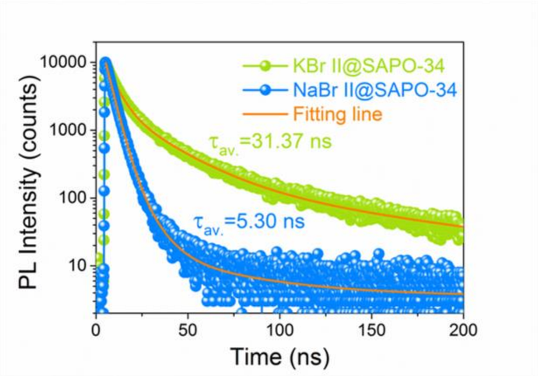

Figure S3. TRPL decays of KBr II@SAPO-34 and NaBr II@SAPO-34.

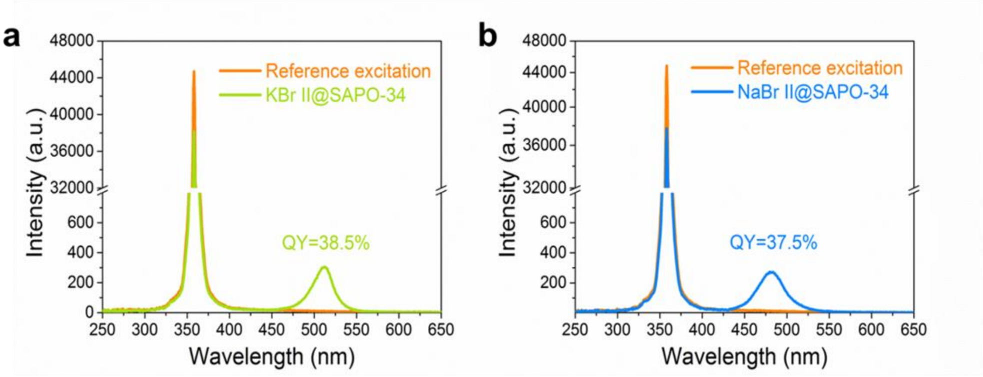

Figure S4. QYs excited at 360 nm for KBr II@SAPO-34 and NaBr II@SAPO-34.

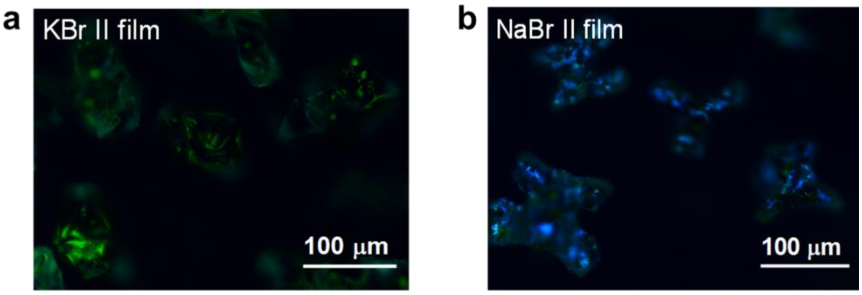

Figure S5. Fluorescence micrographs of KBr II and NaBr II films.

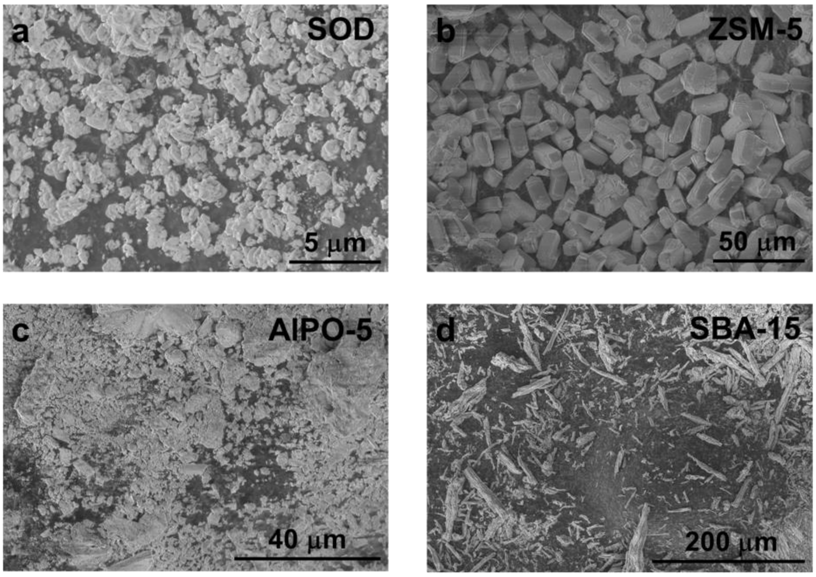

Figure S6. SEM images of pristine micro-/meso-porous materials SOD (a), ZSM-5 (b), AlPO-5 (c) and SBA-15 (d).

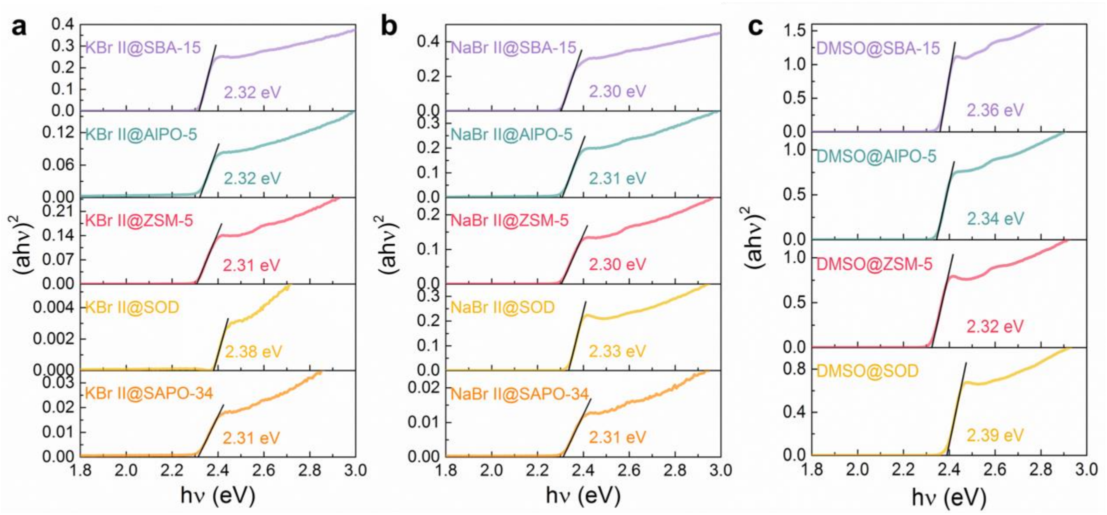

Figure S7. Tauc-plot curves of KBr II, NaBr II and DMSO embedded in porous materials including SAPO-34, SOD, ZSM-5, AlPO-5 and SBA-15.

Table S1. Atomic percentages of the introduced elements in KBr I@SAPO-34, KBr II@SAPO-34, NaBr I@SAPO-34 and NaBr II@SAPO-34 measured by XPS.

composite	K	Na	Cs	Pb	Br	C
**KBr I@SAPO-34**	9.25%			0.46%	9.2%	81.09%
**KBr II@SAPO-34**	4.45%		1.01%	0.28%	4.71%	89.55%
**NaBr I@SAPO-34**		12.56%		0.31%	13.11%	74.02%
**NaBr II@SAPO-34**		1.87%	0.77%	0.14%	1.96%	95.26%

Table S2. TRPL results of KBr II@SAPO-34 and NaBr II@SAPO-34.

composite	$\tau_{av.}$ (ns)	τ_1 (ns)	Proportion of τ_1	τ_2 (ns)	Proportion of τ_2	τ_3 (ns)	Proportion of τ_3
**KBr II@SAPO-34**	31.37	5.665	37.78%	22.671	43.48%	103.43	18.74%
**NaBr II@SAPO-34**	5.30	3.8335	86.10%	9.221	12.07%	47.0	1.83%
